# Number of Patients Studied Prior to Approval of New Medicines: A Database Analysis

**DOI:** 10.1371/journal.pmed.1001407

**Published:** 2013-03-19

**Authors:** Ruben G. Duijnhoven, Sabine M. J. M. Straus, June M. Raine, Anthonius de Boer, Arno W. Hoes, Marie L. De Bruin

**Affiliations:** 1 Utrecht Institute for Pharmaceutical Sciences, Utrecht University, Utrecht, the Netherlands; 2 Medicines Evaluation Board, Utrecht, the Netherlands; 3 Department of Medical Informatics, Erasmus Medical Center, Rotterdam, the Netherlands; 4 Medicines and Healthcare products Regulatory Agency, London, United Kingdom; 5 Julius Center for Health Sciences and Primary Care, University Medical Center Utrecht, Utrecht, the Netherlands; Australian National University, Australia

## Abstract

**Background:**

At the time of approval of a new medicine, there are few long-term data on the medicine's benefit–risk balance. Clinical trials are designed to demonstrate efficacy, but have major limitations with regard to safety in terms of patient exposure and length of follow-up. This study of the number of patients who had been administered medicines at the time of medicine approval by the European Medicines Agency aimed to determine the total number of patients studied, as well as the number of patients studied long term for chronic medication use, compared with the International Conference on Harmonisation's E1 guideline recommendations.

**Methods and Findings:**

All medicines containing new molecular entities approved between 2000 and 2010 were included in the study, including orphan medicines as a separate category. The total number of patients studied before approval was extracted (main outcome). In addition, the number of patients with long-term use (6 or 12 mo) was determined for chronic medication. 200 unique new medicines were identified: 161 standard and 39 orphan medicines. The median total number of patients studied before approval was 1,708 (interquartile range [IQR] 968–3,195) for standard medicines and 438 (IQR 132–915) for orphan medicines. On average, chronic medication was studied in a larger number of patients (median 2,338, IQR 1,462–4,135) than medication for intermediate (878, IQR 513–1,559) or short-term use (1,315, IQR 609–2,420). Safety and efficacy of chronic use was studied in fewer than 1,000 patients for at least 6 and 12 mo in 46.4% and 58.3% of new medicines, respectively. Among the 84 medicines intended for chronic use, 68 (82.1%) met the guideline recommendations for 6-mo use (at least 300 participants studied for 6 mo and at least 1,000 participants studied for any length of time), whereas 67 (79.8%) of the medicines met the criteria for 12-mo patient exposure (at least 100 participants studied for 12 mo).

**Conclusions:**

For medicines intended for chronic use, the number of patients studied before marketing is insufficient to evaluate safety and long-term efficacy. Both safety and efficacy require continued study after approval. New epidemiologic tools and legislative actions necessitate a review of the requirements for the number of patients studied prior to approval, particularly for chronic use, and adequate use of post-marketing studies.

*Please see later in the article for the Editors' Summary*

## Abstract 2

In an evaluation of medicines approved by the European Medicines Agency 2000 to 2010, Ruben Duijnhoven and colleagues find that the number of patients evaluated for medicines approved for chronic use are inadequate for evaluation of safety or long-term efficacy.

## Editors' Summary

**Background:**

Before any new medicine is marketed for the treatment of a human disease, it has to go through extensive laboratory and clinical research. In the laboratory, scientists investigate the causes of diseases, identify potential new treatments, and test these interventions in disease models, some of which involve animals. The safety and efficacy of potential new interventions is then investigated in a series of clinical trials—studies in which the new treatment is tested in selected groups of patients under strictly controlled conditions, first to determine whether the drug is tolerated by humans and then to assess its efficacy. Finally, the results of these trials are reviewed by the government body responsible for drug approval; in the US, this body is the Food and Drug Administration, and in the European Union, the European Medicines Agency (EMA) is responsible for the scientific evaluation and approval of new medicines.

**Why Was This Study Done?:**

Clinical trials are primarily designed to test the efficacy—the ability to produce the desired therapeutic effect—of new medicines. The number of patients needed to establish efficacy determines the size of a clinical trial, and the indications for which efficacy must be shown determine the trial's duration. However, identifying adverse effects of drugs generally requires the drug to be taken by more patients than are required to show efficacy, so the information about adverse effects is often relatively limited at the end of clinical testing. Consequently, when new medicines are approved, their benefit–risk ratios are often poorly defined, even though physicians need this information to decide which treatment to recommend to their patients. For the evaluation of risk or adverse effects of medicines being developed for chronic (long-term) treatment of non-life-threatening diseases, current guidelines recommend that at least 1,000–1,500 patients are exposed to the new drug and that 300 and 100 patients use the drug for six and twelve months, respectively, before approval. But are these guidelines being followed? In this database analysis, the researchers use data collected by the EMA to determine how many patients are exposed to new medicines before approval in the European Union and how many are exposed for extended periods of time to medicines intended for chronic use.

**What Did the Researchers Do and Find?:**

Using the European Commission's Community Register of Medicinal Products, the researchers identified 161 standard medicines and 39 orphan medicines (medicines to treat or prevent rare life-threatening diseases) that contained new active substances and that were approved in the European Union between 2000 and 2010. They extracted information on the total number of patients studied and on the number exposed to the medicines for six months and twelve months before approval of each medicine from EMA's European public assessment reports. The average number of patients studied before approval was 1,708 for standard medicines and 438 for orphan medicines (marketing approval is easier to obtain for orphan medicines than for standard medicines to encourage drug companies to develop medicines that might otherwise be unprofitable). On average, medicines for chronic use (for example, asthma medications) were studied in more patients (2,338) than those for intermediate use such as anticancer drugs (878), or short-term use such as antibiotics (1,315). The safety and efficacy of chronic use was studied in fewer than 1,000 patients for at least six and twelve months in 46.4% and 58.4% of new medicines, respectively. Finally, among the 84 medicines intended for chronic use, 72 were studied in at least 300 patients for six months, and 70 were studied in at least 100 patients for twelve months.

**What Do These Findings Mean?:**

These findings suggest that although the number of patients studied before approval is sufficient to determine the short-term efficacy of new medicines, it is insufficient to determine safety or long-term efficacy. Any move by drug approval bodies to require pharmaceutical companies to increase the total number of patients exposed to a drug, or the number exposed for extended periods of time to drugs intended for chronic use, would inevitably delay the entry of new products into the market, which likely would be unacceptable to patients and healthcare providers. Nevertheless, the researchers suggest that a reevaluation of the study size and long-term data requirements that need to be met for the approval of new medicines, particularly those designed for long-term use, is merited. They also stress the need for continued study of both the safety and efficacy of new medicines after approval and the importance of post-marketing studies that actively examine safety issues.

**Additional Information:**

Please access these websites via the online version of this summary at http://dx.doi.org/10.1371/journal.pmed.1001407.

- The European Medicines Agency (EMA) provides information about all aspects of the scientific evaluation and approval of new medicines in the European Union; its European public assessment reports are publicly available
- The European Commission's Community Register of Medicinal Products is a publicly searchable database of medicinal products approved for human use in the European Union
- The US Food and Drug Administration provides information about drug approval in the US for consumers and for health professionals
- The US National Institutes of Health provides information (including personal stories) about clinical trials

## Introduction

Clinical studies conducted during the development of new medicines are generally designed to show efficacy under strict conditions and are performed in relatively small and selected patient populations [1],[2]. The total number of patients exposed to a new drug before approval is generally assumed to be approximately 1,000 patients [1],[3]. However, to our knowledge, a scientific review of the number of patients studied before European approval has never been conducted. The number of patients exposed to a medicine during trials before approval directly defines the level of knowledge about the efficacy and adverse effects of the new medicine in humans. If few patients have used the medicine before approval, limited information on adverse effects will be available, and the benefit–risk balance is hard to determine. For physicians and other healthcare providers, it is of major importance to provide evidence-based care in everyday practice, but they are often not aware of these limitations. Therefore, the numbers of patients studied before approval merits study, with particular attention to medicines for chronic use.

Although guidelines on the number of patients to be studied are in place, there are no formal European Union requirements for study size and length of follow-up in studies prior to the approval of new medicines. The size of an individual study and the total clinical development programme are mainly, if not entirely, driven by the statistical power needed to establish efficacy. The duration of trials is also determined by the indication for which efficacy must be proven and is rarely continued longer than strictly needed.

For the safety evaluation of medicines developed for chronic treatment of non-life-threatening diseases, the European Medicines Agency (EMA) and its United States counterpart, the Food and Drug Administration, use guidance on patient exposure and the length of time participants are studied based on the International Conference on Harmonisation (ICH) E1 guideline [4],[5]. The E1 guideline sets recommendations on three levels: a total patient exposure of at least 1,000 to 1,500 patients, 6 mo of use by 300 patients, and 12 mo of use by 100 patients [4]. The reasons for choosing 300 and 100 patients as the target numbers to be studied for 6 and 12 mo are not provided in the ICH E1 guideline.

The aim of this study was to review the number of patients exposed to new medicines before approval in the EU (main outcome), with a special focus on long-term exposure for medicines intended for chronic use.

## Methods

The publicly available Community Register of Medicinal Products of the European Commission was used to identify all products approved in the EU through the “centralised procedure” between 1 January 2000 and 31 December 2010, including those that were subsequently withdrawn or suspended [6]. We included all unique, new active substances that were approved in this period. Duplicate products were excluded. Duplicates were defined as all medicines with an identical active substance, and with the same dossier and the same preclinical and clinical studies, but with two or more product names (e.g., Januvia and Xelevia).

European public assessment reports (EPARs) are publicly available on the EMA's website [7]. From the EPARs for all products, we extracted the total number of participants in the studies (patients as well as healthy volunteers) who received at least one dose of the medicine. Data were read from automated records of the European Commission [6] directly, and additional data (number of participants) were extracted by R. G. D. These data were systematically checked by M. L. D. B. to ensure accuracy or to resolve uncertainties if numbers were not reported clearly.

The intended use of medications was assessed based on the official indication at approval. With this indication as a reference, intended treatment duration was classified as chronic, intermediate, or short term by R. G. D. and M. L. D. B. Any discrepancies were resolved in discussion with A. d. B. Examples of chronic use included asthma and HIV medication, intermediate length of use included anticancer treatment, and short-term use included antimicrobial medication and most analgesics and diagnostic agents.

For all medicines intended for chronic use we extracted additional information on the number of patients who had received treatment for at least 6 mo and at least 12 mo. If no (reliable) information on the number of exposed patients could be obtained, patient exposure was categorised as missing. In some EPARs the number of patients treated with the study medication for 12 mo was reported, whereas use for at least 6 mo was not reported. In such cases the number of participants with 12-mo use was imputed as 6-mo use.

In addition, information was obtained on special authorisation status (orphan status, exceptional circumstances, and conditional approval) where applicable. Products were categorised as orphan medicines if the EMA's Committee for Orphan Medicinal Products had granted them official EU orphan status; all other medicines were categorised as “standard medicines”.

Based on the total number of patients exposed before approval, all products were divided into one of the following five groups: less than 500 patients, 500 to 1,000 patients, 1,000 to 2,000 patients, 2,000 to 5,000 patients, and more than 5,000 patients. To assess long-term use before approval, the numbers of participants studied for at least 6 mo and for at least 12 mo were calculated. The cutoff values used for the number of patients required in long-term studies were chosen according to the clinical safety guideline: at least 300 for 6-mo use and at least 100 for 12-mo use [4],[5].

The non-parametric Wilcoxon two-sample test was used to determine whether there was a statistically significant difference in the number of participants studied for medicines still on the market versus those withdrawn from the market as of 4 November 2011.

## Results

We identified 200 newly approved medicines in the period 2000–2010, of which 161 were standard (non-orphan) medicines (80.5%) and 39 were official orphan medicines (19.5%). The specific medicines and number of patients studied are listed in Dataset S1.

### Total Number of Patients Studied

**Figure 1:**
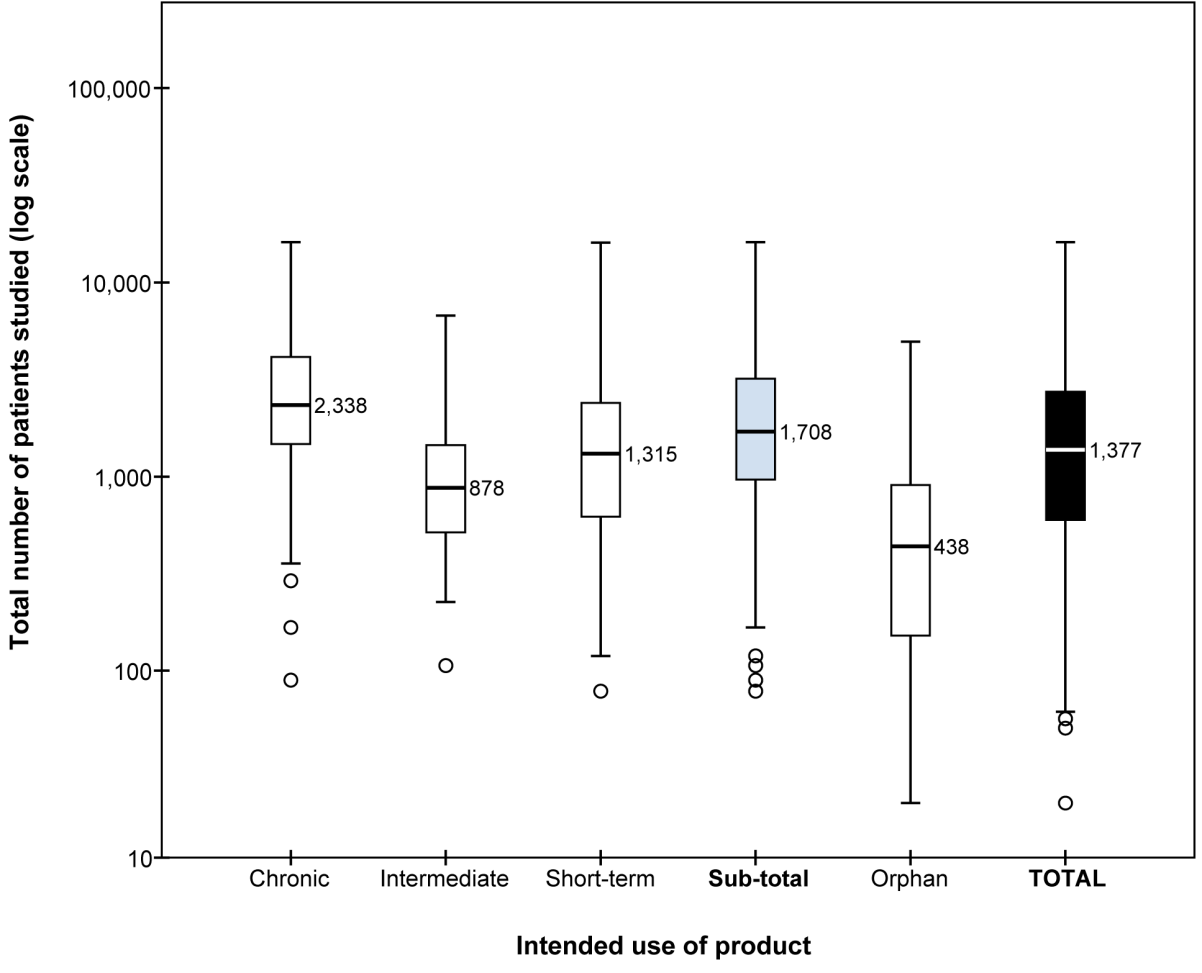
Boxplots with medians of the number of patients studied before approval. Results for standard (non-orphan) medicines are presented by intended length of use of the products (chronic, intermediate, or short-term) and as one group (sub-total). Boxplots present the 50th percentile, i.e., the median value is given, with the interquartile range (25th and 75th percentiles) indicated by the box, the 2nd and 98th percentiles indicated by the horizontal bars of the whiskers, and outliers indicated by individual circles. The total number of patients studied (*y*-axis) is plotted on a logarithmic scale.

The median number of total patients studied per medicine was 1,708 (interquartile range [IQR] 968–3,195) for standard medicines and 438 (IQR 132–915) for orphan medicines (Figure 1).

Orphan medicines generally had small numbers of patients in clinical studies; 31 (79.5%) of the products had been used by fewer than 1,000 patients. Eight orphan medicines had been tested in more than 1,000 patients (plerixafor, mecasermin, rufinamide, trabectedin, sorafenib, ziconotide, anagrelide, and imatinib).

Among the standard medicines, 90 (55.9%) of the 161 products had been studied in fewer than 2,000 patients in total, of which 20 (13.7%) were studied in fewer than 500. 52 (32.3%) products were studied in 2,000–5,000 patients, and 19 (11.8%) were studied in more than 5,000 (Table 1).

**Table 1:** Number (percent) of medicines categorised according to total number of individuals studied prior to marketing.

Total Number of Patients	Standard Medicines				Orphan Medicines	Total
	Chronic	Intermediate	Short-Term	Sub-Total		
<500	6/84 (7.1%)	6/27 (22.2%)	8/50 (16.0%)	20/161 (12.4%)	21/39 (53.8%)	41/200 (20.5%)
500–1,000	4/84 (4.8%)	9/27 (33.3%)	10/50 (20.0%)	23/161 (14.3%)	10/39 (25.6%)	33/200 (16.5%)
1,000–2,000	23/84 (27.4%)	10/27 (37.0%)	14/50 (28.0%)	47/161 (29.2%)	7/39 (17.9%)	54/200 (27.0%)
2,000–5,000	38/84 (45.2%)	1/27 (3.7%)	13/50 (26.0%)	52/161 (32.3%)	1/39 (2.6%)	53/200 (26.5%)
>5,000	13/84 (15.5%)	1/27 (3.7%)	5/50 (10.0%)	19/161 (11.8%)	0/39 (0.0%)	19/200 (9.5%)
Total	84/84 (100%)	27/27 (100%)	50/50 (100%)	161/161 (100%)	39/39 (100%)	200/200 (100%)

Percentages are column percentages.

The number of patients receiving medicines for short-term treatment before marketing authorisation varied considerably. Eight (16.0%) medicines had been studied in fewer than 500 patients, whereas five (10.0%) medicines had been studied in more than 5,000 patients, and 13 (26.0%) in 2,000–5,000 patients. Medicines intended for intermediate length of use were tested on the smallest number of patients before approval; 25 (92.4%) medicines were used by fewer than 2,000 patients. Within this category, 20 (74.1%) medicines were indicated for treatment of cancer. Medicines for chronic use were studied in larger numbers of patients during clinical development. In total, 51 (60.7%) of these products had been used by 2,000 or more patients, of which 13 (15.1%) had been studied in more than 5,000 patients.

Six medicines in our analyses had their marketing authorisation subsequently suspended or withdrawn. Medicines still on the market had been studied before approval in a median 1,694 patients (IQR 899–3,167), versus 2,161 patients (IQR 968–5,479) for suspended or withdrawn medicines; this difference was not statistically significant (*p* = 0.61; Wilcoxon two-sample test).

### Long-Term Studies of Medicines for Chronic Use

**Figure 2:**
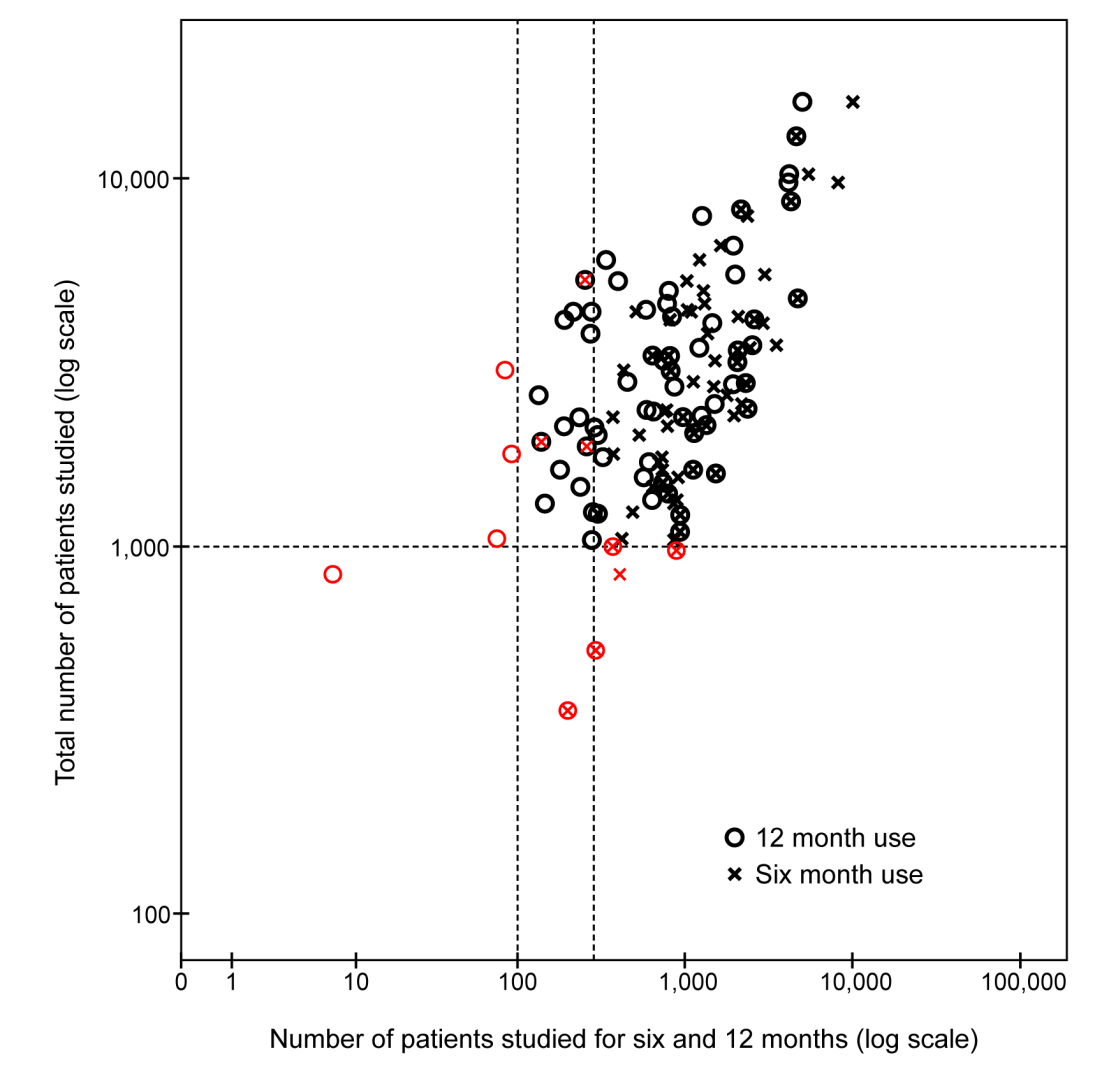
Scatterplot displaying the total number of patients studied before approval plotted against the number of patients studied long term (for 6 and 12 mo) for chronic medication. Reference lines are added to indicate the minimum criteria from the ICH E1 guideline: 1,000 patients in total and 300 and 100 patients studied for 6 and 12 mo, respectively. Any products not meeting the ICH E1 guideline recommendations are shown in red.

Among the 84 medicines intended for chronic use, 69 (82.1%) met the patient exposure recommendations for 6-mo use (at least 300 participants studied for 6 mo and at least 1,000 participants in total), and 67 (79.8%) of the medicines met the criteria for 12-mo patient exposure (at least 100 participants) (Table 2; Figure 2).

**Table 2:** Number (percent) of medicines categorised according to total number of individuals studied for 6 and 12 mo (long term) prior to marketing.

Total Number of Patients		Number of Patients with 6-mo Use				Number of Patients with 12-mo Use			
		<300	300–1,000	>1,000	Missing	<100	100–1,000	>1,000	Missing
<1,000	(*n* = 10)	**3/10 (30.0%)**	**3/10 (30.0%)**	**0/10 (0.0%)**	4/10 (40.0%)	**1/10 (10.0%)**	**3/10 (30.0%)**	**0/10 (0.0%)**	6/10 (60.0%)
1,000–5,000	(*n* = 61)	**2/61 (3.3%)**	30/61 (49.2%)	28/61 (45.9%)	1/61 (1.6%)	**3/61 (4.9%)**	39/61 (63.9%)	16/61 (26.2%)	3/61 (4.9%)
>5,000	(*n* = 13)	**1/13 (7.7%)**	0/13 (0.0%)	11/13 (84.6%)	1/13 (7.7%)	**0/13 (0.0%)**	3/13 (23.1%)	9/13 (69.2%)	1/13 (7.7%)
Total	(*n* = 84)	**6/84 (7.1%)**	33/84 (39.3%)	39/84 (46.4%)	6/84 (7.1%)	**4/84 (4.8%)**	45/84 (53.6%)	25/84 (29.8%)	10/84 (11.9%)

Percentages presented are row percentages for 6 and 12 mo use. Products with (1) a total number of patients studied of fewer than 1,000, (2) fewer than 300 studied for 6 mo, or (3) fewer than 100 studied for 12 mo do not meet the guideline criteria, and are shown in bold. For purposes of calculation and display, missing data were assumed to be in compliance with the recommended patient exposures.

Safety and efficacy of chronic use were studied in fewer than 1,000 individuals for 6 mo or more in 41 (48.8%) medicines, and for 12 mo or more in 49 (58.3%) medicines.

Six (7.1%) medicines had been used by fewer than 300 patients for a minimum of 6 mo. 33 (39.3%) medicines had been tested in 300 to 1,000 patients, and 39 (46.4%) medicines had been tested in more than 1,000 patients, both for a minimum of 6 mo. For six (7.1%) medicines, information on the number of patients included in long-term studies was missing in the EPAR.

For 45 (53.6%) medicines, 100 to 1,000 patients had been studied for at least 12 mo, and 25 (29.8%) products had been studied in over 1,000 patients. For four medicines (4.8%), fewer than 100 patients had been studied for at least 12 mo. Data on 12-mo use was missing in 10 (11.9%) EPARs.

## Discussion

To our knowledge no recent research has systematically assessed the number of patients and volunteers exposed to new medicines before approval. A previous study of product licence applications in the UK between 1987 and 1989 by Rawlins and Jefferys showed that the median number of individuals exposed to new active substances in premarketing studies was 1,480 (range 129–9,400) for successful applications, and 1,052 (range 43–15,962) for unsuccessful applications [8]. The proportion of withdrawals after approval in our study was comparable to that in the previous UK study. In contrast to the study by Rawlins and Jefferys, our study was restricted to successful applications, but study size has increased only marginally since the late 1980s. For both policy makers and healthcare providers, it is important to be aware of the inherent limitations of the size of trials conducted before approval with regard to efficacy as well as adverse effects.

The aim of the ICH E1 guideline on data requirements for medicines for long-term use is to assure at least a minimum of experience and knowledge of long-term efficacy and safety before approval. Overall, 1,000 to 1,500 patients in total, and a minimum of 300 and 100 treated for at least 6 and 12 mo, respectively, are required. Results from our study show that the minimal requirements are met by approximately 80% of new medicines approved for chronic use in the EU.

Although increasing the number of patients exposed to a medicine before approval could be justified, especially for medicines intended for long-term use, the requirement could delay new products entering the market. In the current era, in which patients and healthcare providers demand more rapid access to new medicines, this would not be acceptable for most stakeholders in the field. Furthermore, randomised controlled trials sufficiently large to accurately assess long-term safety and effectiveness are expensive, and epidemiological studies in the post-marketing phase may be more suitable.

As an example, in the meta-analysis by Nissen and Wolski published in 2007 on myocardial infarction in users of rosiglitazone, myocardial infarction incidence was approximately 36 per 10,000 patients in the control group, with rosiglitazone use incurring an estimated increased risk (odds ratio) of 1.43 [9]. This meta-analysis contributed much to the US Food and Drug Administration's decision to update the labelling and restrict the prescription of rosiglitazone-containing products [10]. In the EU, it led the EMA to formally suspend the marketing authorisation of rosiglitazone-containing products.

For a clinical trial to reveal a relative risk of 1.43 with statistical significance (a power of 80% and α of 0.05%, see Table 3), approximately 30,000 patients per study arm would be required, necessitating a total study size of 60,000 patients. Such large trials would be difficult to conduct before approval, and meta-analyses or observational studies are more feasible for studying such outcomes.

**Table 3:** Sample sizes (number of study participants) required to detect adverse effects of medicines in trials and cohort studies (with required number per study arm and assuming a significance level of 95% [α = 0.05] and power of 80% [β = 0.2]).

Relative Risk	Incidence of the Outcome in the Study	
	1∶5,000	1∶1,000
2.0	117,697	23,511
2.5	61,025	12,187
3.0	39,228	7,832
5.0	14,707	2,934
7.5	7,888	1,572
10	5,323	1,059

However, for chronic medications, the clinical safety guidelines require too few patients to be studied long term. The possibility of detecting long-term adverse events from follow-up of only 300 patients for 6 mo or 100 patients for 12 mo, as required in the current ICH E1 guideline, is insufficient. It would be more sensible to move towards a minimum targeted long-term study size of 1,000 to 1,500 patients, comparable to the overall study size now required [4].

Knowledge of a medicine's benefit–risk profile, including its effectiveness in clinical practise and associated adverse effects, should only increase over time and with increasing use. Clinical use outside the restrictive environment of trials may be the only way to achieve a full understanding of the safety profile [3],[11]–[13]. Pharmacovigilance activities involving active monitoring of spontaneous reporting systems, registries, post-marketing safety studies, and risk management plans (RMPs), have proven to be important tools in facilitating this process [14],[15]. Spontaneous adverse event reporting has been the main source of information in pharmacovigilance for decades, but it has important limitations. To be of value, spontaneous reporting requires healthcare providers and patients to notice and report the adverse effect [1]. This is possible for rare adverse effects, but cannot be done for common morbidities with a long time to disease onset [1],[15]–[19]. New methods are employed in the US Food and Drug Administration Sentinel Initiative [20],[21] and EU EU-ADR project [22] to signal such adverse effects and address these problems. Both projects aim to use anonymised automated healthcare records to continuously monitor medicines for the disproportionate occurrence of adverse events [23]–[25].

RMPs have become an important tool to progressively extend the knowledge of the safety of newly approved medicines [26],[27]. International guidance on RMPs has been established (ICH E2E guideline “Pharmacovigilance Planning”) [28] and adopted in European law in 2005 [29]. With RMPs, pharmacovigilance has passed a turning point, moving from a largely reactive role to a continuous proactive risk management approach. Now, the demonstration of safety in practice and the process of filling in gaps in knowledge after marketing have been added to the passive monitoring of case reports [15]. An RMP serves as the central document in pharmacovigilance activities for an individual product, and contains three elements: (1) a safety specification describing the potential and identified risks as well as important missing information on adverse effects, (2) the pharmacovigilance plan, which describes proposals to acquire more data on possible risks, identified risks, and missing information, and (3) the risk minimisation plan [28],[30]. RMPs are prepared and maintained by the pharmaceutical companies, but require approval by regulatory authorities, who may require companies to add new risks to the RMP or to initiate new risk minimisation activities, including new studies for safety or efficacy. The newest EU legislation requires a summary of the RMP to be made public [30].

Post-marketing observational pharmacoepidemiological studies are essential, even though confounding in observational data may be impossible to eliminate completely. RMPs and other pharmacovigilance activities do not overcome the problems due to insufficient statistical power and the need for large study sizes to detect less common adverse effects in clinical trials (as discussed above for the case of rosiglitazone). Signals of adverse effects require formal and adequately powered observational studies before the issue can be quantified and addressed.

Regulator-driven post-marketing studies are possible in both the US [31],[32] and EU (called post-authorisation safety studies in RMPs) [33],[34]. In the US, the effectiveness of post-marketing studies was reviewed several years ago, and the review indicated that pharmaceutical companies often progress slowly if at all in initiating, continuing, and completing such studies [15],[16]. For the EU situation, such a detailed review has not been conducted, but a review by the EMA itself indicated that studies progressed well [35]. However, this review considered the initiation of a study as progress, rather than considering how much time was spent before finalisation. The European pharmacovigilance legislation adopted in December 2010 provides an important new legal basis to overcome these problems and makes it possible to impose requirements for post-authorisation safety studies on pharmaceutical companies when needed [33],[34],[36].

Recently, new approaches in regulation have been proposed by means of “adaptive licensing” [37],[38]. In adaptive licensing, the regulatory outcome (either rejection or approval of a new medicine) is changed to a process in which requirements for first approval are less strict, but research must continue after approval, and marketing authorisation continuation is dependent on the results. Such an approach could solve limitations in current regulatory practice, as it is expected to provide better data on product effectiveness in real world clinical practice, rather than only efficacy in clinical trials [39]. Furthermore, observational studies on adverse effects could then become a formal part of the approval dossier. In addition to classical observational study designs, new study methods could provide new tools to further analyse safety [40].

A re-evaluation of the requirements regarding study size and long-term data for approval of new medicines seems to be merited. Such a discussion should involve healthcare providers, patients, and academia, as well as industry and regulators, and should include debate on the level of acceptable uncertainty, especially for adverse events and the long-term outcomes for chronic medication.

The numbers of individuals studied before approval of new medicines in Europe from 2000 to 2010 are comparable to the study sizes for medicines approved in UK in the 1980s, and are generally adequate to assess only short-term efficacy. For most approved medicines intended for chronic use, the number of patients studied before marketing is insufficient to study safety and long-term efficacy. In light of new scientific and legislative tools to monitor benefits and risks in clinical use, discussion of the long-term exposure requirements for approval of medicines, particularly for medicines intended for chronic use, seems warranted.

## Supporting Information

Dataset S1 **Overview of all medicines included.** Overview and details of the medicines included in the study.
(PDF)

## Abbreviations

EMA: European Medicines Agency
EPAR: European public assessment report
ICH: International Conference on Harmonisation
IQR: interquartile range
RMP: risk management plan
