# Supplementary material for: Number of Patients Studied Prior to Approval of New Medicines: A Database Analysis
Source: PLoS Med. 2013 Mar 19;10(3):e1001407. doi: 10.1371/journal.pmed.1001407 (PMC3601954; doi:10.1371/journal.pmed.1001407)
Supplement: Dataset S1 — Overview of all medicines included. Overview and details of the medicines included in the study. (PDF) [file pmed.1001407.s001.pdf]

Categorisation of all products according to approved indication

Supplement to: Duijnhoven RG, Straus SMJM, Raine JM, de Boer A, Hoes AW, et al. (2013) *Number of Patients Studied Prior to Approval of New Medicines: A Database Analysis* . **PLoS Med** 10(3): e1001407. doi:10.1371/journal.pmed.1001407

							No. of patients studied:			
**Year of approval**	**Orphan**	**International proprietary name**	**International non-proprietary name**	**ATC code**	**Use**	**Indication**	**Total**	**6 months**	**12 months**	**Withdrawn/ Suspended**
2010	No	**Brilique**	**ticagrelor**	B01AC24	*Chronic*	Peripheral Vascular Diseases, Acute Coronary Syndrome	9,733	8,219	4,147	No
2010	No	**Daxas**	**roflumilast**	R03DX07	*Chronic*	Chronic Obstructive Pulmonary Disease	6,563	1,639	1,953	No
2010	No	**Prolia**	**denosumab**	M05BX04	*Chronic*	Osteoporosis, Postmenopausal Bone Resorption	13,000	4,625	4,625	No
2010	No	**Sycrest**	**asenapine**	N05AH05	*Chronic*	Bipolar Disorder	4,565	1,314	785	No
2010	**Yes**	**Arzerra**	**ofatumumab**	L01XC10	*Orphan (intermediate)*	Chronic Lymphocytic B-Cell Leukemia	648	N/A	N/A	No
2010	No	**Brinavess**	**vernakalant**	C01BG11	*Short-term*	Atrial Fibrillation	883	N/A	N/A	No
2010	No	**Elonva**	**corifollitropin alfa**	G03GA09	*Short-term*	Reproductive Techniques, Assisted Ovulation Induction	1,088	N/A	N/A	No
2010	No	**Rapiscan**	**regadenoson**	C01EB21	*Short-term*	Myocardial Perfusion Imaging	1,651	N/A	N/A	No
2010	**Yes**	**Revolade**	**eltrombopag**	B02BX05	*Orphan (chronic)*	Idiopathic Thrombocytopenic Purpura	422	192	41	No
2010	No	**Ruconest**	**conestat alfa**	B06AC04	*Short-term*	Angio-oedema (hereditary)	119	N/A	N/A	No
2010	No	**Scintimun**	**technetium antigranulocyte antibody (besilesomab)**	V09HA03	*Short-term*	Radionuclide Imaging, Osteomyelitis	1,311	N/A	N/A	No
2010	No	**Silodyx**	**Silodosin**	G04CA04	*Intermediate*	Prostatic Hyperplasia	1,581	961	438	No
2010	No	**Votrient**	**pazopanib**	L01XE11	*Intermediate*	Renal Cell Carcinoma	1,645	326	185	No
2010	**Yes**	**Vpriv**	**velaglucerase alfa**	A16AB10	*Orphan (chronic)*	Gaucher Disease	94	52	10	No
2009	No	**Cimzia**	**certolizumab pegol**	L04AB05	*Chronic*	Rheumatoid Arthritis	2,367	2,367	2,367	No
2009	No	**Conbriza**	**bazedoxifene**	G03XC02	*Chronic*	Postmenopausal Osteoporosis	4,720	4,720	4,720	No
2009	No	**Efient**	**prasugrel**	B01AC22	*Chronic*	Unstable Angina, Acute Coronary Syndrome, Myocardial Infarction	8,656	4,300	4,300	No
2009	No	**Fablyn**	**lasofoxifene**	G03XC03	*Chronic*	Postmenopausal Osteoporosis	10,257	5,474	4,201	No
2009	No	**Multaq**	**dronedarone**	C01BD07	*Chronic*	Atrial Fibrillation	3,410	2,073	2,073	No
2009	No	**Onglyza**	**saxagliptin**	A10BH03	*Chronic*	Diabetes Mellitus Type 2	4,042	2,934	1,459	No
2009	No	**Oslif Breezhaler**	**indacaterol**	R03AC18	*Chronic*	Chronic Obstructive Pulmonary Disease	6,003	1,226	339	No
2009	No	**Resolor**	**prucalopride**	A03AE04	*Chronic*	Constipation	2,717	1,490	869	No
2009	No	**RoActemra**	**tocilizumab**	L04AC07	*Chronic*	Rheumatoid Arthritis, Juvenile Rheumatoid Arthritis	2,439	2,188	1,507	No
2009	No	**Samsca**	**tolvaptan**	C03XA01	*Chronic*	Inappropriate ADH Syndrome	3,294	800	817	No
2009	No	**Simponi**	**golimumab**	L04AB06	*Chronic*	Ankylosing Spondylitis, Rheumatoid Arthritis, Psoriatic Arthritis	2,758	2,343	1,943	No
2009	**Yes**	**Cayston**	**aztreonam**	J01DF01	*Orphan (short-term)*	Respiratory Tract Infections, Cystic Fibrosis	373	N/A	N/A	No
2009	No	**Stelara**	**ustekinumab**	L04AC05	*Chronic*	Psoriasis	2,266	1,970	1,258	No
2009	No	**Thymanax**	**agomelatine**	N06AX22	*Chronic*	Major Depressive Disorder	5,260	1,030	400	No
2009	No	**Vedrop**	**tocofersolan**	A11HA08	*Chronic*	Vitamin E Deficiency, Cholestasis	167	missing	missing	No
2009	No	**ChondroCelect**	**characterised viable autologous cartilage cells expanded *ex vivo* expressing specific marker proteins**	M09AX02	*Short-term*	Cartilage Diseases	463	N/A	N/A	No
2009	No	**Victoza**	**liraglutide**	A10BX07	*Chronic*	Diabetes Mellitus Type 2	4,211	2,085	840	No
2009	No	**Zebinix**	**eslicarbazepine**	N03AF04	*Chronic*	Epilepsy	1,694	700	612	No
2009	No	**ellaOne**	**ulipristal acetate**	G03AD02	*Short-term*	Postcoital Contraception	3,560	N/A	N/A	No
2009	**Yes**	**Firdapse**	**amifampridine**	N07XX05	*Orphan (chronic)*	Lambert-Eaton Myasthenic Syndrome	282	missing	missing	No
2009	No	**Firmagon**	**degarelix**	L02BX02	*Intermediate*	Prostatic Neoplasms	1,974	1,148	1,148	No

2009	Yes	**Ilaris**	**canakinumab**	L04AC08	*Orphan (chronic)*	Cryopyrin-associated Periodic Syndromes	104	62	56	No
2009	No	**Iressa**	**gefitinib**	L01XE02	*Intermediate*	Non-Small-Cell Lung Carcinoma	1,855	N/A	N/A	No
2009	No	**Javlor**	**vinflunine**	L01CA05	*Short-term*	Transitional Cell Carcinoma, Urologic Neoplasms	1,203	N/A	N/A	No
2009	Yes	**Mepact**	**mifamurtide**	L03AX15	*Orphan (intermediate)*	Osteosarcoma	248	N/A	N/A	No
2009	Yes	**Mozobil**	**plerixafor**	L03AX16	*Orphan (short-term)*	Multiple Myeloma, Hematopoietic Stem Cell Transplantation, Lymphoma	1,161	N/A	N/A	No
2009	Yes	**Nplate**	**romiplostim**	B02BX04	*Orphan (chronic)*	Idiopathic Thrombocytopenic Purpura	271	153	114	No
2009	No	**Removab**	**catumaxomab**	L01XC09	*Intermediate*	Ascites, Cancer	270	N/A	N/A	No
2008	No	**Bridion**	**sugammadex**	V03AB35	*Short-term*	Neuromuscular Blockade	1,833	N/A	N/A	No
2008	No	**Adenuric**	**febuxostat**	M04AA03	*Chronic*	Gout	3,521	3,521	2,531	No
2008	No	**Doribax**	**doripenem**	J01DH04	*Short-term*	Cross Infection, Bacterial Infections, Pneumonia, Bacterial, Pneumonia, Ventilator-Associated, Urinary Tract Infections	3,207	N/A	N/A	No
2008	No	**Intelence**	**etravirine**	J05AG04	*Chronic*	HIV Infections	1,041	861	279	No
2008	No	**Ranexa**	**ranolazine**	C01EB18	*Chronic*	Angina Pectoris	3,463	2,451	1,223	No
2008	No	**Vimpat**	**lacosamide**	N03AX18	*Chronic*	Epilepsy	1,338	898	638	No
2008	Yes	**Firazyr**	**icatibant**	C01EB19	*Orphan (short-term)*	Hereditary Angioedemas	962	N/A	N/A	No
2008	Yes	**Kuvan**	**sapropterin**	A16AX07	*Orphan (chronic)*	Phenylketonurias	647	missing	missing	No
2008	No	**Mycamine**	**micafungin**	J02AX05	*Short-term*	Candidiasis	3,028	N/A	N/A	No
2008	No	**Pradaxa**	**dabigatran etexilate**	B01AE07	*Short-term*	Replacement Arthroplasty, Venous Thromboembolism	6,976	N/A	N/A	No
2008	No	**Relistor**	**methylnaltrexone bromide**	A06AH01	*Short-term*	Constipation, Opioid-Related Disorders	566	N/A	N/A	No
2008	No	**Tyverb**	**lapatinib**	L01XE07	*Intermediate*	Breast Neoplasms	1,149	N/A	N/A	No
2008	Yes	**Volibris**	**ambrisentan**	C02KX02	*Orphan (chronic)*	Pulmonary Hypertension	725	627	514	No
2008	No	**Xarelto**	**rivaroxaban**	B01AX06	*Short-term*	Replacement Arthroplasty, Venous Thromboembolism	9,550	N/A	N/A	No
2007	No	**Altargo**	**retapamulin**	D06AX13	*Short-term*	Impetigo, Staphylococcal Skin Infections	2,150	N/A	N/A	No
2007	Yes	**Atriance**	**nelarabine**	L01BB07	*Orphan (intermediate)*	Precursor T-Cell Lymphoblastic Leukemia-Lymphoma	459	N/A	N/A	No
2007	No	**Circadin**	**melatonin**	N05CH01	*Intermediate*	Sleep Initiation and Maintenance Disorders	1,361	373	146	No
2007	Yes	**Diacomit**	**stiripentol**	N03AX17	*Orphan (chronic)*	Juvenile Myoclonic Epilepsy	740	80	80	No
2007	No	**Ecalta**	**anidulafungin**	J02AX06	*Short-term*	Candidiasis	204	N/A	N/A	No
2007	Yes	**Elaprase**	**idursulfase**	A16AB09	*Orphan (chronic)*	Mucopolysaccharidosis II	187	missing	missing	No
2007	No	**Celsentri**	**maraviroc**	J05AX09	*Chronic*	HIV Infections	840	410	7	No
2007	No	**Galvus**	**vildagliptin**	A10BH02	*Chronic*	Diabetes Mellitus Type 2	3,784	1,370	274	No
2007	No	**Invega**	**paliperidone**	N05AX13	*Chronic*	Psychotic Disorders, Schizophrenia	2,326	754	652	No
2007	No	**Isentress**	**raltegravir**	J05AX08	*Chronic*	HIV Infections	899	800	missing	No
2007	No	**Lucentis**	**ranibizumab**	S01LA04	*Chronic*	Wet Macular Degeneration, Macular Edema, Diabetes Complications	1,096	937	936	No
2007	No	**Orencia**	**abatacept**	L04AA24	*Chronic*	Rheumatoid Arthritis, Juvenile Rheumatoid Arthritis	2,778	2,311	2,311	No
2007	No	**Prezista**	**darunavir**	J05AE10	*Chronic*	HIV Infections	1,783	375	92	No
2007	No	**Rasilez**	**aliskiren**	C09XA02	*Chronic*	Hypertension	7,896	2,367	1,270	No
2007	No	**Sebivo**	**telbivudine**	J05AF11	*Chronic*	Hepatitis B, Chronic	1,491	743	743	No
2007	Yes	**Increlex**	**mecasermin**	H01AC03	*Orphan (chronic)*	Growth, Laron Syndrome	1,516	76	76	No

2007	Yes	Inovelon	rufinamide	N03AF03	*Orphan (chronic)*	Epilepsy	1,978	1,236	922	No
2007	No	Toviaz	fesoterodine	G04BD11	*Chronic*	Overactive Urinary Bladder	1,789	missing	missing	No
2007	No	Xelevia	sitagliptin	A10BH01	*Chronic*	Type 2 Diabetes Mellitus	4,393	1,038	588	No
2007	No	Nevanac	nepafenac	S01BC10	*Short-term*	Ophthalmologic Surgical Procedures, Postoperative Pain	1,938	N/A	N/A	No
2007	No	Optimark	gadoversetamide	V08CA06	*Short-term*	Magnetic Resonance Imaging	2,398	N/A	N/A	No
2007	Yes	Revlimid	lenalidomide	L04AX04	*Orphan (intermediate)*	Multiple Myeloma	353	248	163	No
2007	Yes	Soliris	eculizumab	L04AA25	*Orphan (chronic)*	Paroxysmal Hemoglobinuria	716	184	97	No
2007	Yes	Tasigna	nilotinib	L01XE08	*Orphan (intermediate)*	Chronic Myelogenous Leukemia, BCR-ABL Positive	438	310	173	No
2007	Yes	Torisel	temsirolimus	L01XE09	*Orphan (intermediate)*	Renal Cell Carcinoma, Mantle-Cell Lymphoma	915	103	N/A	No
2007	No	Vectibix	panitumumab	L01XC08	*Intermediate*	Colorectal Neoplasms	1,304	N/A	N/A	No
2007	Yes	Yondelis	trabectedin	L01CX01	*Orphan (intermediate)*	Ovarian Neoplasms, Sarcoma	1,018	N/A	N/A	No
2006	No	Champix	varenicline	N07BA03	*Intermediate*	Tobacco Use Cessation	6,739	456	112	No
2006	No	Cubicin	daptomycin	J01XX09	*Short-term*	Gram-Positive Bacterial Infections, Soft Tissue Infections, Bacterial Endocarditis, Bacteremia	1,474	N/A	N/A	No
2006	Yes	Evoltra	clofarabine	L01BB06	*Orphan (intermediate)*	Precursor Cell Lymphoblastic Leukemia-Lymphoma	132	N/A	N/A	No
2006	Yes	Exjade	deferasirox	V03AC03	*Orphan (chronic)*	Beta-Thalassemia, Iron Overload	652	456	missing	No
2006	No	Gardasil	papillomavirus (human types 6, 11, 16, 18) papillomavirus (human types 16, 18)	J07BM01	*Short-term*	Condylomata Acuminata, Uterine Papillomavirus Infections, Cervical Dysplasia Immunization	16,014	N/A	N/A	No
**2006**	**No**	Acomplia	rimonabant	A08AX01	*Chronic*	Obesity	16,120	10,062	5,023	Yes
2006	No	Baraclude	entecavir	J05AF10	*Chronic*	Hepatitis B	1,392	796	796	No
2006	No	Byetta	exenatide	A10BX04	*Chronic*	Type 2 Diabetes Mellitus	2,997	825	825	No
2006	No	Kiovig	Immunoglobulins, normal human, for intravascular administration	J06BA02	*Intermediate*	Immunologic Deficiency Syndromes, Guillain-Barre Syndrome, Bone Marrow Transplantation, Idiopathic Thrombocytopenic Purpura, Mucocutaneous Lymph Node Syndrome	106	N/A	N/A	No
2006	No	Macugen	pegaptanib	S01LA03	*Chronic*	Wet Macular Degeneration	975	892	892	No
2006	No	Neupro	rotigotine	N04BC09	*Chronic*	Restless Legs Syndrome, Parkinson Disease	2,008	535	302	No
2006	No	Preotact	parathyroid hormone	H05AA03	*Chronic*	Postmenopausal Osteoporosis	3,167	2,059	2,058	No
2006	No	Tysabri	natalizumab	L04AA23	*Chronic*	Multiple Sclerosis	1,617	1,123	1,123	No
2006	No	Luminity	microspheres of phospholipids	V08DA04	*Short-term*	Echocardiography	2,526	N/A	N/A	No
2006	Yes	Myozyme	aglucosidase alfa	A16AB07	*Orphan (chronic)*	Glycogen Storage Disease Type II	61	50	35	No
2006	Yes	Naglazyme	galsulfase	A16AB08	*Orphan (chronic)*	Mucopolysaccharidosis VI	56	missing	missing	No
2006	Yes	Nexavar	sorafenib	L01XE05	*Orphan (intermediate)*	Renal Cell Carcinoma, Hepatocellular Carcinoma	1,957	253	42	No
2006	Yes	Sprycel	dasatinib	L01XE06	*Orphan (intermediate)*	Chronic Myelogenous Leukemia, BCR-ABL Positive, Precursor Cell Lymphoblastic Leukemia-Lymphoma	511	56	N/A	No
2006	No	Sutent	sunitinib	L01XE04	*Intermediate*	Neuroendocrine Tumors, Gastrointestinal Stromal Tumors, Renal Cell Carcinoma	876	334	63	No
**2006**	Yes	Thelin	sitaxentan	**C02KX03**	*Orphan (chronic)*	**Pulmonary Hypertension**	**899**	**526**	**378**	**Yes**
2006	No	Tygacil	tigecycline	J01AA12	*Short-term*	Soft Tissue Infections, Bacterial Infections, Bacterial Skin Diseases	1,415	N/A	N/A	No
2005	No	Aloxi	palonosetron	A04AA05	*Short-term*	Vomiting - Cancer	2,348	N/A	N/A	No

2005	No	**Avastin**	**bevacizumab**	L01XC07	*Intermediate*	Non-Small-Cell Lung Carcinoma, Colorectal Neoplasms, Renal Cell Carcinoma, Breast Neoplasms	1,032	N/A	154	No
2005	No	**Kepivance**	**palifermin**	V03AF08	*Short-term*	Mucositis	786	N/A	N/A	No
2005	No	**Aptivus**	**tipranavir**	J05AE09	*Chronic*	HIV Infections	3,195	1,514	748	No
2005	No	**Azilect**	**rasagiline**	N04BD02	*Chronic*	Parkinson Disease	1,453	645	238	No
2005	No	**Procoralan**	**ivabradine**	C01EB17	*Chronic*	Angina Pectoris	4,340	513	216	No
2005	No	**Xolair**	**omalizumab**	R03DX05	*Chronic*	Asthma	5,300	254	254	No
2005	No	**Noxafil**	**posaconazole**	J02AC04	*Short-term*	Aspergillosis, Coccidioidomycosis, Candidiasis, Mycoses	925	109	27	No
2005	**Yes**	**Prialt**	**ziconotide**	N02BG08	*Orphan (short-term)*	Spinal Injections, Pain	1,048	N/A	153	No
2005	No	**Tarceva**	**erlotinib**	L01XE03	*Intermediate*	Non-Small-Cell Lung Carcinoma, Pancreatic Neoplasms	759	N/A	N/A	No
2005	No	**Vasovist**	**gadofosveset**	V08CA11	*Short-term*	Magnetic Resonance Angiography	1,438	N/A	N/A	No
2004	No	**Alimta**	**pemetrexed**	L01BA04	*Intermediate*	Non-Small-Cell Lung Carcinoma, Mesothelioma	226	N/A	N/A	No
2004	No	**Angiox**	**bivalirudin**	B01AE06	*Short-term*	Transluminal Percutaneous Coronary Angioplasty, Acute Coronary Syndrome	15,817	N/A	N/A	No
2004	No	**Erbitux**	**cetuximab**	L01XC06	*Intermediate*	Colorectal Neoplasms, Head and Neck Neoplasms	522	N/A	N/A	No
2004	No	**Faslodex**	**fulvestrant**	L02BA03	*Intermediate*	Breast Neoplasms	1,559	423	N/A	No
2004	No	**Abilify**	**aripiprazole**	N05AX12	*Chronic*	Bipolar Disorder, Schizophrenia	4,947	1,293	805	No
2004	No	**Apidra**	**insulin glulisine**	A10AB06	*Chronic*	Diabetes Mellitus	1,617	736	180	No
2004	No	**Ariclaim**	**duloxetine**	N06AX21	*Chronic*	Diabetic Neuropathies	4,127	818	191	No
2004	No	**Cholestagel**	**colesevelam**	C10AC04	*Chronic*	Hypercholesterolemia	1,870	260	260	No
2004	No	**Emselex**	**darifenacin**	G04BD10	*Chronic*	Urge Urinary Incontinence, Overactive Urinary Bladder	7,257	missing	missing	No
2004	No	**Levemir**	**insulin detemir**	A10AE05	*Chronic*	Diabetes Mellitus	3,159	1,732	missing	No
2004	No	**Lyrica**	**pregabalin**	N03AX16	*Chronic*	Epilepsy, Anxiety Disorders, Neuralgia	8,228	2,164	2,163	No
2004	No	**Mimpara**	**cinacalcet**	H05BX01	*Chronic*	Parathyroid Neoplasms, Hypercalcemia, Hyperparathyroidism	1,749	732	325	No
2004	No	**Protelos**	**strontium ranelate**	M05BX03	*Chronic*	Postmenopausal Osteoporosis	4,138	2,600	2,600	No
2004	No	**Raptiva**	**efalizumab**	L04AA21	*Chronic*	Psoriasis	3,014	433	84	Yes
2004	No	**Reyataz**	**atazanavir**	J05AE08	*Chronic*	HIV Infections	2,244	373	235	No
2004	No	**Telzir**	**fosamprenavir**	J05AE07	*Chronic*	HIV Infections	1,000	372	372	No
2004	No	**TachoSil**	***combinations***	B02BC30	*Short-term*	Surgical Hemostasis	215	N/A	N/A	No
2004	No	**Velcade**	**bortezomib**	L01XX32	*Intermediate*	Multiple Myeloma	256	N/A	N/A	No
2004	**Yes**	**Xagrid**	**anagrelide**	L01XX35	*Orphan (chronic)*	Essential Thrombocythemia	4,955	4,955	4,955	No
2003	**Yes**	**Aldurazyme**	**laronidase**	A16AB05	*Orphan (chronic)*	Mucopolysaccharidosis I	68	10	10	No
2003	**Yes**	**Carbaglu**	**carglumic acid**	A16AA05	*Orphan (chronic)*	Carbamoyl-Phosphate Synthase I Deficiency Disease, Amino Acid Metabolism, Inborn Errors, Propionic Acidemia	20	20	15	No
2003	No	**Emend**	**aprepitant**	A04AD12	*Short-term*	Postoperative Nausea and Vomiting, VomitingCancer	3,335	N/A	N/A	No
2003	No	**Emtriva**	**emtricitabine**	J05AF09	*Chronic*	HIV Infections	2,136	1,348	1,348	No
2003	No	**Forsteo**	**teriparatide**	H05AA02	*Chronic*	Postmenopausal Osteoporosis, Osteoporosis	2,032	1,137	1,137	No
2003	No	**Fuzeon**	**enfuvirtide**	J05AX07	*Chronic*	HIV Infections	1,541	913	569	No
2003	No	**Hepsera**	**adefovir dipivoxil**	J05AF08	*Chronic*	Chronic Hepatitis B	522	294	294	No
2003	No	**Humira**	**adalimumab**	L04AB04	*Chronic*	Ankylosing Spondylitis, Rheumatoid Arthritis, Crohn Disease, Psoriatic Arthritis, Juvenile Rheumatoid	2,575	1,785	134	No
2003	No	**Vivanza**	**vardenafil**	G04BE09	*Intermediate*	Erectile Dysfunction	4,413	1,630	730	No

2002	No	**Arixtra**	**fondaparinux**	B01AX05	*Short-term*	Venous Thrombosis, Pulmonary Embolism, Unstable Angina, Myocardial Infarction	2,294	N/A	N/A	No
2002	No	**Cialis**	**tadalafil**	G04BE08	*Intermediate*	Pulmonary Hypertension	1,112	720	231	No
2002	No	**Dynastat**	**parecoxib**	M01AH04	*Short-term*	Postoperative Pain	3,550	N/A	N/A	No
2002	**Yes**	**Glivec**	**imatinib**	L01XE01	*Orphan (intermediate)*	BCR-ABL Positive Chronic Myelogenous Leukemia, Dermatofibrosarcoma, Gastrointestinal Stromal Tumors, Myelodysplastic-Myeloproliferative Diseases, Precursor Cell Lymphoblastic Leukemia-Lymphoma, Hypereosinophilic Syndrome	1,027	N/A	9	No
2002	No	**InductOs**	**dibotermin alfa**	M05BC01	*Short-term*	Tibial Fractures, Spinal Fusion, Internal Fracture Fixation	635	N/A	N/A	No
2002	No	**Invanz**	**ertapenem**	J01DH03	*Short-term*	Streptococcal Infections, Surgical Wound Infection, Gram-Negative Bacterial Infections, Community-Acquired Infections, Staphylococcal Infections, Bacterial Pneumonia	2,100	N/A	N/A	No
2002	No	**Neulasta**	**pegfilgrastim**	L03AA13	*Intermediate*	Cancer, Neutropenia	465	N/A	N/A	No
**2002**	**No**	**Dynepo**	**epoetin delta**	B03XA	*Chronic*	Anemia, Chronic Kidney Failure	1,308	861	146	Yes
2002	No	**Evra**	**progestogens and estrogens, fixed combinations**	G03AA	*Chronic*	Contraception	3,300	643	643	No
2002	No	**Kineret**	**anakinra**	L04AC03	*Chronic*	Rheumatoid Arthritis	1,240	490	283	No
2002	No	**Lumigan**	**bimatoprost**	S01EE03	*Chronic*	Open-Angle Glaucoma, Ocular Hypertension	1,708	957	missing	No
2002	No	**Viread**	**tenofovir disoproxil**	J05AF07	*Chronic*	Chronic Hepatitis B, HIV Infections	1,050	422	75	No
2002	**Yes**	**Somavert**	**pegvisomant**	H01AX01	*Orphan (chronic)*	Acromegaly	241	129	84	No
2002	No	**Tamiflu**	**sseltamivir**	J05AH02	*Short-term*	Influenza, Human	7,642	N/A	N/A	No
2002	**Yes**	**Tracleer**	**bosentan**	C02KX01	*Orphan (chronic)*	Pulmonary Hypertension, Systemic Scleroderma	174	100	28	No
2002	No	**Vfend**	**voriconazole**	J02AC03	*Short-term*	Aspergillosis, Candidiasis, Mycoses	1,214	56	N/A	No
2002	No	**Xigris**	**drotrecogin alfa (activated)**	B01AD10	*Short-term*	Sepsis, Multiple Organ Failure	1,058	N/A	N/A	No
2002	**Yes**	**Zavesca**	**miglustat**	A16AX06	*Orphan (chronic)*	Gaucher Disease, Niemann-Pick Diseases	80	75	75	No
2001	No	**Aerius**	**desloratadine**	R06AX27	*Short-term*	Urticaria, Allergic Seasonal Rhinitis, Allergic Perennial Rhinitis	2,346	N/A	N/A	No
2001	No	**Cancidas**	**caspofungin**	J02AX04	*Short-term*	Candidiasis, Aspergillosis	623	N/A	N/A	No
2001	**Yes**	**Fabrazyme**	**agalsidase beta**	A16AB04	*Orphan (chronic)*	Fabry Disease	73	missing	missing	No
2001	No	**Fasturtec**	**rasburicase**	V03AF07	*Short-term*	Hyperuricemia	375	N/A	N/A	No
2001	No	**Foscan**	**temoporfin**	L01XD05	*Intermediate*	Squamous Cell Carcinoma, Head and Neck Neoplasms	855	N/A	N/A	No
2001	No	**INOmax**	**nitric oxide**	R07AX01	*Short-term*	Pulmonary Hypertension, Respiratory Insufficiency	206	N/A	N/A	No
2001	No	**Ketek**	**telithromycin**	J01FA15	*Short-term*	Community-Acquired Infections, Pharyngitis, Sinusitis, Bacterial Pneumonia, Chronic Bronchitis, Tonsillitis	2,485	N/A	N/A	No
2001	No	**MabCampath**	**alemtuzumab**	L01XC04	*Intermediate*	Chronic Lymphocytic B-Cell Leukemia	996	N/A	N/A	No
2001	No	**Metalyse**	**tenecteplase**	B01AD11	*Short-term*	Myocardial Infarction	543	N/A	N/A	No
2001	No	**NeuroBloc**	**botulinum toxin**	M03AX01	*Intermediate*	Torticollis	596	N/A	N/A	No
2001	No	**Osigraft**	**eptotermin alfa**	M05BC02	*Short-term*	Tibial Fractures	500	N/A	N/A	No
2001	No	**Ovitrelle**	**chorionic gonadotrophin**	G03GA01	*Short-term*	Female Infertility, Assisted Reproductive Techniques, Anovulation	535	N/A	N/A	No
2001	Yes	**Replagal**	**agalsidase alfa**	A16AB03	*Orphan (chronic)*	Fabry Disease	50	16	missing	No
2001	No	**Aranesp**	**darbepoetin alfa**	B03XA02	*Chronic*	Anemia, Chronic Kidney Failure, Cancer	1,578	1,534	1,534	No
2001	No	**SonoVue**	**sulfur hexafluoride**	V08DA05	*Short-term*	Echocardiography, Ultrasonography	1,319	N/A	N/A	No

2001	No	**Ceprotin**	**protein C**	B01AD12	*Chronic*	Protein C Deficiency, Purpura Fulminans	89	missing	missing	No
2001	No	**Rapamune**	**sirolimus**	L04AA10	*Chronic*	Kidney Transplantation, Graft Rejection	2,247	976	976	No
2001	No	**Starlix**	**nateglinide**	A10BX03	*Chronic*	Type 2 Diabetes Mellitus	2,122	789	190	No
2001	No	**Targretin**	**bexarotene**	L01XX25	*Intermediate*	Lymphoma, T-Cell, Cutaneous	513	91	N/A	No
2001	No	**Travatan**	**travoprost**	S01EE04	*Chronic*	Open-Angle Glaucoma, Ocular Hypertension	1,925	139	139	No
2001	No	**Xeloda**	**capecitabine**	L01BC06	*Intermediate*	Colorectal Neoplasms, Colonic Neoplasms, Stomach Neoplasms, Breast Neoplasms	603	550	8	No
2000	No	**DaTSCAN**	**Iodine ioflupane (123I)**	V09AB03	*Short-term*	Single-Photon Emission-Computed Tomography, Lewy Body Disease, Parkinson Disease, Alzheimer Disease	454	N/A	N/A	No
2000	No	**Luveris**	**lutropin alfa**	G03GA07	*Short-term*	Ovulation Induction, Female Infertility	78	N/A	N/A	No
**2000**	**No**	**NeoSpect**	**technetium (99mTc) depreotide**	V09IA05	*Short-term*	Radionuclide Imaging	968	N/A	N/A	Yes
2000	No	**Orgalutran**	**ganirelix**	H01CC01	*Short-term*	Assisted Reproductive Techniques, Ovulation Induction	1,217	N/A	N/A	No
2000	No	**Actos**	**pioglitazone**	A10BG03	*Chronic*	Type 2 Diabetes Mellitus	4,339	1,095	278	No
2000	No	**Thyrogen**	**thyrotropin**	H01AB01	*Intermediate*	Thyroid Neoplasms	419	N/A	N/A	No
**2000**	**No**	**Agenerase**	**amprenavir**	**J05AE05**	*Chronic*	**HIV Infections**	**358**	**200**	**200**	**Yes**
2000	No	**Tractocile**	**atosiban**	G02CX01	*Short-term*	Premature Birth	1,260	N/A	N/A	No
**2000**	**No**	**Avandia**	**rosiglitazone**	A10BG02	*Chronic*	Type 2 Diabetes Mellitus	5,479	3,000	2,000	Yes
2000	No	**Azopt**	**brinzolamide**	S01EC04	*Chronic*	Ocular HypertensionGlaucoma, Open-Angle	1,227	303	303	No
2000	No	**Enbrel**	**etanercept**	L04AB01	*Chronic*	Ankylosing Spondylitis, Rheumatoid Arthritis, Psoriatic Arthritis, Psoriasis, Juvenile Rheumatoid Arthritis	2,801	1,126	455	No
2000	No	**Keppra**	**levetiracetam**	N03AX14	*Chronic*	Epilepsy	2,349	780	592	No
2000	No	**Lantus**	**insulin glargine**	A10AE04	*Chronic*	Diabetes Mellitus	2,106	1,104	289	No
2000	No	**NovoMix**	**insulin aspart**	A10AD05	*Chronic*	Diabetes Mellitus	291	missing	missing	No
2000	No	**Visudyne**	**verteporfin**	S01LA01	*Intermediate*	Degenerative Myopia, Macular Degeneration	878	207	191	No
2000	No	**Panretin**	**alitretinoin**	L01XX22	*Chronic*	HIV Infections, Kaposi Sarcoma	469	missing	missing	No
2000	No	**PegIntron**	**peginterferon alfa-2b**	L03AB10	*Chronic*	Chronic Hepatitis C	1,219	940	940	No
2000	No	**Renagel**	**sevelamer**	V03AE02	*Chronic*	Hyperphosphatemia, Renal Dialysis	384	192	missing	No

N/A: long-term use is not applicable

* regular (non-orphan) medicines for chronic use, not meeting the ICH E1 criteria of 1000 users in total and 300 subjects exposed for six months and 100 for 12 months are **marked red**

* orphan medicines are **marked blue**

This is a supplement to an open-access article distributed under the terms of the Creative Commons Attribution License, which permits unrestricted use, distribution, and reproduction in any medium, provided the original author and source are credited.
